# Vascular Injury in Elective Anterior Surgery of the Lumbar Spine: A Narrative Review

**DOI:** 10.7759/cureus.20267

**Published:** 2021-12-08

**Authors:** Eleni Pappa, Dimitrios Stergios Evangelopoulos, Ioannis S Benetos, Spiridon Pneumaticos

**Affiliations:** 1 5th Orthopaedic Department, KAT General Hospital, Athens, GRC; 2 3rd Orthopaedic Department, KAT General Hospital, National and Kapodistrian University of Athens School of Medicine, Athens, GRC; 3 Orthopaedics, University of Athens, KAT Trauma Hospital, Athens, GRC; 4 3rd Orthopaedic Department, KAT General Hospital, Athens, GRC

**Keywords:** vascular complications, orthopaedics, spine surgery, anterior lumbar spine surgery, vascular injury

## Abstract

The incidence of anterior lumbar surgery is increasing as the population is aging. Although adverse events regarding vasculature injury are uncommon, several have been described in the current literature. Complications can be categorized based on the time of occurrence, more specifically intraoperative or postoperative, but also regarding the nature of vascular damage such as thrombosis, occlusion, or rupture. The rate of complications is higher in the setting of revision anterior surgery than with primary anterior lumbar surgery. Moreover, the incidence of revision anterior surgery is also increasing in contrast to the past. Through this narrative review, an effort is made for a thorough understanding of the complications associated with anterior lumbar surgery, which will aid in the prevention, recognition, and management of this rare complication.

## Introduction and background

Access to the anterior lumbar spine is often required for the treatment of many issues such as infection, deformity, tumor, trauma, and degenerative disease. This approach is preferred, as it leads to a successful discectomy, the ability to implant high-profile internal fixation devices, thorough debridement and irrigation of necrotic tissues, removal of migrated or spinal devices, and for interbody fusion. Nowadays, with increasing rates of low back pain and the breakthrough of total disc arthroplasty (TDA) in addition to prosthetic disc nucleus replacement, access to the anterior lumbar spine is gaining ground in spinal surgery, as described by recent literature [1].

During anterior spinal exposure of the levels L4-L5 and L5-S1, the left common iliac vessels need to be mobilized, as they course obliquely across the anterior aspect of the L5 body and traverse portions of the L4-L5 and L5-S1 disc spaces. The left common iliac vein (CIV) is the most dorsally located and is the most likely vascular structure to be injured during anterior lumbar spinal surgery, as indicated by the published case series. However, thrombosis of the iliac artery, accompanied by thrombectomy and arterial repair, has also been reported at an incidence of 0.45%. Moreover, a case of rupture of the descending thoracic aorta at the site of retraction during corpectomy, resulting in the death of the patient, has also been highlighted in the literature [2].

The purpose of this narrative review is to underline the incidence of major vascular injury during anterior spinal surgery, to report predisposing risk factors, and to discuss management techniques.

## Review

A left-sided, paramedian muscle-preserving approach is frequently used for both the single and multilevel approaches of the lumbar spine. During the specific approach, the left rectus abdominis muscle is retracted laterally. For approaches involving the levels L2-L3 and above, a muscle-splitting approach through the left flank is usually performed, with or without thoracotomy and diaphragm mobilization, depending on the cephalic extent of the spinal reconstruction [3]. The ascending iliolumbar vein is routinely ligated during exposures in the L4-L5 disc space but not during isolated L5-S1 disc space exposures. The significance of exposure and extensive mobilization of the left iliac vessels, in order to establish retraction to the right side of the spine during exposure of the L4-L5 disc space, is highlighted in recent case series. Ligation of the L4 segmental vessels has also been reported, especially during exposure of the L4-L5 disc space. Regarding retractor systems, both self-retaining and automatic retractor systems have been used, however, hand-held retraction seems to be superior concerning the manipulation and control of the major blood vessels (i.e., aorta, superior and inferior vena cavae, iliac artery, and vein). What is highlighted in most of the case series studies is that a team approach is usually preferred, with the spine surgeon and vascular surgeon present in the operating room. In any case of vascular repair, it was performed by a vascular surgeon [4].

Vascular complications

As mentioned in the current literature, a major vascular injury is defined as an injury to the iliac vessels, vena cava, and aorta. Most CIV injuries occur at levels L4-L5.

Risk factors are usually present in patients with CIV injuries, including previous osteomyelitis or discogenic infection, previous anterior spinal surgery spondylolisthesis, large anterior osteophyte, transitional lumbosacral vertebra, or anterior migration of the interbody device. Regarding arterial injury, it usually occurs at the terminal aorta where the L3-L4 level is situated, which is usually the site of enlarged lateral osteophytes, at an incidence of approximately 0.01%-0.06% [5].

Vascular injury is likely to take place either during initial spinal exposure or during various forms of spinal reconstruction, including corpectomy, distraction, or osteotomy. In all cases, mobilization of major vascular structures led to the injury, which was entrapped in an extensive inflammatory reaction, as a result of the conditions identified above. In the most recent study of Ho et al., both venous injury and venous thrombosis were mentioned by 6.5% and 23%, respectively. Additionally, when vascular injury and postoperative thrombosis are present, both the length of stay and procedure time are elongated, however, no statistically significant difference has been mentioned in the literature [6].

Regarding iliac venous injury, first, bleeding control was achieved with compression proximal and distal to venotomy, using hemostatic agents such as a sponge stick and/or Kitner dissector. In those cases, suction is usually avoided so as to decrease the extension of the venotomy and vascular clamps are rarely applied. When a vascular injury is present, the patient positioning preferred is Trendelenburg in order to decrease the venous pressure in the lower extremities. Definitive repair is attempted when the anesthesia organizes fluid resuscitation, including volume recovery or transfusion.

Postoperative magnetic resonance venography (MRV) is an essential tool to demonstrate vascular patency for cases of intraoperative vascular injury.

Regarding the management of vascular injury, the primary venous repair was achieved by lateral venorrhaphy with 4-0 or 5-0 prolene stitches. Also, topical agents can be used to reinforce incomplete repairs. These topical agents included those enhancing clot formation, and hemostatic agents, including porcine gelatine and oxidized cellulose, which are the most commonly used. In cases of venotomy, vascular clips have also been used, reinforced by the existing topical agents above. Venous thrombosis can also complicate suture repair of the left CIV, where the diagnosis can be made by postoperative MRV. In such cases, the inferior vena cava filter (IVC) has been placed successfully [7-8].

Discussion

As the population is aging, the need for anterior or posterior spinal surgery procedures is also increasing. Furthermore, the frequency of revision surgery is also expected to be increasing as well, leading to an increased risk for major vascular injury. The incidence of vascular injury in anterior spinal surgery is characterized by a variety of frequencies in the published case series.

In the current literature, a high incidence has been reported, as in the case of Baker et al., who reported 15.4% of vascular injuries in their case series. Fantini et al., on the other hand, also reported an extremely low 2.7% [7,9].

It is quite noticeable in the literature that the majority of vascular injuries take place in the iliac venous system, during the exposure of L4-L5 and L5-S1, so it is likely that studies including higher exposure may report lower rates of vascular injury [10].

Moreover, the inability of the surgeon to mobilize the iliac vessels, which is quite often in cases of atherosclerotic vessels, is also likely to increase the risk of vascular damage, as the vessels' elasticity is diminished, however, no statistical difference has been stated. In such cases, preoperative radiographic planning of the vasculature should make the spine surgeon suspicious of any likely danger in vessel mobilization during anterior surgery. Even in the absence of atherosclerotic plaques, soft atherosclerotic tissues may be present, which may rupture and lead to vessel occlusion and thrombosis.

Regarding arterial damage and thrombosis, the rates presented in the literature are quite low, as 0.45% for thrombosis and 2.4% for arterial injury are reported. Most of these cases happened due to fixed retraction on the iliac vessels, including mechanically fixed retractors and Steinman pins. Both of these manipulations have been abandoned and nowadays the iliac vessels are retracted only by hand, at intervals of 20 minutes to decrease any incidence of thrombosis, as mentioned in the majority of current studies [11-12].

Furthermore, we should always take into account any predisposing risk factors, which are like to lead to vascular injury. Spondylolisthesis, anterior osteophytes, previous spinal infection, such as tuberculosis, revision surgery for migrating spinal devices, as well as previous anterior spine surgery, are all identified as predisposing conditions to vascular damage. This can be explained by the fact that all of the situations above lead to local perivertebral tissue inflammation resulting in the lack of mobility of the surrounding vessels during manipulation. Subsequently, preoperative planning with 3D CT angiography is a gold standard technique to study the perivertebral vascular anatomy. Kiyohara et al. introduced the anatomical evaluation of lumbar vessels via MRI preoperatively, in order to diminish any vascular damage intraoperatively, as the lumbar arteries are not always located in the center of the vertebra, while Karhade et al. used intraoperative natural language processing (NLP) algorithms for vascular injury from free-text operative notes for the prediction of vascular injury, with quite promising results.

Additionally, the presence of anatomic anomalies is also a risk factor for vascular injury during anterior surgery of the lumbar spine [13]. On the one hand, segmentation anomalies, including the transitional lumbosacral vertebra, lead to the fact that the last mobile segment lies dorsally to the iliac vein or inferior vena cava (IVC), which mandates an approach to the last mobile segment, which is lateral to the left common iliac vessels, making the division of the ascending iliolumbar vessels essential in order to gain adequate exposure. Anatomic variations may also include the local vessels, such as left-sided or duplicated IVC, retro-aortic course of the left renal vein, as well as variations of the lumbosacral junction [14].

The management of vascular damage is a procedure that can be life-saving, such as in the case of iliac vein rupture, where a simple vascular occlusion can lead to acceptable outcomes regarding blood loss. In the case of a non-feasible suture of the vascular wall, hemostatic agents can also be used to preserve the hemodynamic stability of the patient. Lateral aortorrhaphy with a single 4-0 prolene suture placed in a figure-of-eight fashion is characterized as successful, and aortic clamping was also not necessary. No instances of arterial thrombotic complications are mentioned in the literature. What is outlined in most of the studies is that even in the slightest manipulation of the iliac vein system, thrombosis and occlusion of the vessels may be precipitated. This may not manifest any symptoms during the early postoperative period where the patient is non-ambulatory. In order to assess the possibility of iliac vein thrombosis and occlusion, duplex ultrasonography of the lower limb is essential. However, this method is not predicting any thrombosis above the inguinal ligament, so a negative test may not eliminate thrombosis of the ipsilateral iliac system. This is where MRV and IVC are the gold standards for the detection of pelvic vein thrombosis, in cases of iliac vein repair after anterior spine surgery. In the case of a proximal vein thrombosis, an IVC filter is then placed [15-16].

When patients are likely to undergo revision surgery due to anterior migration of internal devices, such as interbody fusion or TDA of L4-L5 or L5-S1, preoperative planning with 3D reconstruction angiography is essential in order to assess the relationship of the local major vessels with the hardware. An interesting finding is the tending or compression of the left common iliac vein, which usually predicts the need for IVC filter placement for occult venous thromboembolism prevention. The draping of the left common iliac vein around migrated hardware is a common finding that makes the dissection of the vessel difficult due to the scar formation, which limits the vessel mobilization. This is the case where the preoperative placement of stent graft within the left CIV could overcome the possibility of major hemorrhage intraoperative leading to acute occlusion of the left CIV and increased morbidity [17-18].

However, it is quite highlighted in the current literature that in cases of anterior spinal surgery that are accompanied by predisposing risk factors such as the above, detailed preoperative planning is essential in order to diminish any possibility of fatal vascular damage. Also, it is quite clear in recent studies, that the presence of vascular injuries and postoperative venous thrombosis leads to increased transfusion rates, morbidity, and in-hospital mortality, as highlighted by Goot et al., where the most cases of injury signed in the left common iliac vein (LCIV) [19-20]. On the other hand, Chiriano et al. reported that by anterior retroperitoneal access to the lumbar spine (ARES), the frequency of vascular injury in cases involving the disc of L4-L5 was higher in comparison with the other levels while CIV laceration was associated with higher rates of postoperative deep vein thrombosis [21].

## Conclusions

Anterior lumbar spine surgery is accompanied by an elevated risk of major vasculature injury, including rupture, hemorrhage, or thrombosis. As the population is aging, the need for revision surgery of the anterior lumbar spine is also increasing, leading to a higher incidence of vasculature injury. The spine surgeon should be suspicious of any predisposing factors as the aforementioned and be familiar with exquisite preoperative planning, especially in cases of revision surgery. Knowledge of techniques of intraoperative repair of hemorrhage, as well as the management of thrombosis, is important, together with the aid of a specialized vascular team, which will all lead to better treatment and the uneventful discharge for all the patients undergoing anterior lumbar spine surgery. However, more studies need to be established in the future, in order to fully understand the mechanism of vessel ruptures during anterior lumbar spine surgery.
